# Enhancing the sensitivity of magnetic sensors by 3D metamaterial shells

**DOI:** 10.1038/srep44762

**Published:** 2017-03-17

**Authors:** Carles Navau, Rosa Mach-Batlle, Albert Parra, Jordi Prat-Camps, Sergi Laut, Nuria Del-Valle, Alvaro Sanchez

**Affiliations:** 1 Departament de Física, Universitat Autònoma de Barcelona, 08193 Bellaterra, Barcelona, Catalonia, Spain; 2 Institute for Quantum Optics and Quantum Information of the Austrian Academy of Sciences, A-6020 Innsbruck, Austria; 3 Institute for Theoretical Physics, University of Innsbruck, A-6020 Innsbruck, Austria

## Abstract

Magnetic sensors are key elements in our interconnected smart society. Their sensitivity becomes essential for many applications in fields such as biomedicine, computer memories, geophysics, or space exploration. Here we present a universal way of increasing the sensitivity of magnetic sensors by surrounding them with a spherical metamaterial shell with specially designed anisotropic magnetic properties. We analytically demonstrate that the magnetic field in the sensing area is enhanced by our metamaterial shell by a known factor that depends on the shell radii ratio. When the applied field is non-uniform, as for dipolar magnetic field sources, field gradient is increased as well. A proof-of-concept experimental realization confirms the theoretical predictions. The metamaterial shell is also shown to concentrate time-dependent magnetic fields upto frequencies of 100 kHz.

In the current information age, sensors are key elements that provide the monitoring information essential to our interconnected society. Magnetic sensors are one of the most important components of these technologies^1^,^2^. Computers have large memories that are read by magnetic sensors. Airplanes, cars - particularly, self-driving ones - and other vehicles are routinely running safely thanks to the inputs obtained by complex arrays of magnetic sensors. Factories and even whole cities (particularly, within the concept of smart cities) heavily rely on magnetic sensors to improve efficiency and sustainability. Magnetic sensors play essential roles in space exploration as well^3^.

Different kinds of magnetic sensors exist based on several physical principles^1^,^2^. They include Hall-effect^4^, fluxgate^5^, magnetoresistive of both AMR^6^ and GMR^7^ types, magnetic tunnel junctions^8^, SQUIDs^9^, or recent proposals based on nitrogen vacancies on diamond^10^. A common strategy to improve their sensitivity is using magnetic materials^2^ to concentrate magnetic fields around the sensor, typically by simply placing it in the gap between two high-permeability magnetic material pieces. This has been used for example in Hall-effect sensors^11^,^12^ and in magnetoresistive ones^13^,^14^,^15^.

A specially important branch of applications of magnetic sensors deals with detecting tiny magnetic fields^16^,^17^. The enormous progress in sensor sensitivities has allowed magnetic sensors to be applied to the demanding biomagnetic applications^18^,^19^, including magnetoencephalography^20^ or ultra-low-field magnetic resonance imaging^21^. In these applications, it is common to measure not only the magnetic field but also its spatial gradient in order to help eliminate noise from undesired field sources^17^.

The continuously increasing demand for higher sensitivity of magnetic sensors requires new ideas or solutions. We present in this work a novel way to increase their sensitivity using the special properties for shaping magnetic fields provided by magnetic metamaterials.

Metamaterials, whose electromagnetic properties depend on their internal structure rather than on their chemical constitution, have represented a revolution in the ways of controlling electromagnetic fields^22^,^23^,^24^. Magnetic metamaterials are a particularly useful type^25^,^26^,^27^. They have enabled the realization of novel devices for controlling magnetic fields, including magnetic cloaks or magnetic hoses to transfer fields to long distances^27^,^28^,^29^,^30^,^31^,^32^,^33^,^34^,^35^.

One of the properties that can be enhanced by the use of metamaterials is the concentration of electromagnetic fields^36^. For electromagnetic waves, however, concentration in a volume surrounded by metamaterials is not practical: using transformation optics^22^,^37^ it can be seen that a material is required in the hole of the device, preventing the placement of a sensor^36^. Metamaterials have also been used for concentrating thermal energy^38^,^39^ and acoustic waves^40^,^41^. For the magnetic case, using transformation optics it was shown that significant concentration of magnetic fields can be achieved in an empty region surrounded by a long hollow cylinder placed perpendicular to the field^30^,^31^,^42^. This was done using extreme anisotropic magnetic metamaterials (media with very large permeability in one direction and very small in the perpendicular one). However, a real application to enhance magnetic sensors would require a three-dimensional (3D) spatial concentration, instead of the more idealized studied two-dimensional (2D) geometry of a long cylindrical shell, to take full advantage of the field penetration along all dimensions.

In this work, we analytically show and experimentally demonstrate that a 3D spherical metamaterial shell surrounding a magnetic sensor can concentrate both the magnetic field and gradient in its vicinity to large values and therefore increase its sensitivity. Analytic expressions obtained from Maxwell equations allow to derive the transfer function from the measured to the actual external fields, both in the case of an external uniform field and a dipolar field. We demonstrate how the fully 3D geometry (a spherical shell) is fundamentally different from the 2D case, which results in important advantages. Finally, we show how the ideal metamaterial shells can be discretized in practice into devices composed of soft ferromagnetic (e. g. ferrites or steel) and diamagnetic (e. g. superconducting) components. A proof-of-concept realization of an all-ferromagnetic concentrating shell is constructed to experimentally demonstrate the theoretical results. Experimentally, our device exhibits an almost constant field concentration ratio in a wide frequency range, from 1 Hz to 100 kHz.

## Results

### Spherical concentrators in a uniform applied field

In order to study the magnetic concentration properties of a spherical shell in an applied field, we derive the analytic solutions of the magnetostatic Maxwell equations. Consider a spherical shell of inner radius *R*_1_ and outer radius *R*_2_ made of a linear, homogeneous and anisotropic magnetic material. A uniform magnetic field **H**_0_ is applied in the *z* direction. The shell is characterized by its radial, azimuthal, and polar relative permeabilities, *μ*_*r*_, *μ*_*φ*_, and *μ*_*θ*_, respectively, such that *B*_*r*_ = *μ*_0_*μ*_*r*_*H*_*r*_, *B*_*φ*_ = *μ*_0_*μ*_*φ*_*H*_*φ*_, and *B*_*θ*_ = *μ*_0_*μ*_*θ*_*H*_*θ*_, being *B*_*r,φ,θ*_ and *H*_*r,φ,θ*_ the radial, azimuthal, and polar components of the magnetic induction **B** and the magnetic field **H**, respectively, and *μ*_0_ the permeability of free space. We choose *μ*_*φ*_ = *μ*_*θ*_ for simplicity. By applying magnetostatic boundary conditions, Maxwell equations are analytically solved in all regions of space (see Supplementary Discussion 1 for the full derivation).

The solutions show two important properties. First, the field inside the spherical hole is a uniform field in the direction of the applied magnetic field,


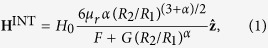


where 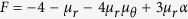, 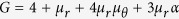, and 
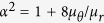
. Second, the field created by the shell at the external region is the field of a dipole located at the shell center, pointing to the direction of the applied magnetic field with magnetic moment


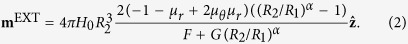


### Non-distortion field cases

For the 2D case of an infinitely long hollow cylinder in a transverse applied magnetic field *H*_0_ it was demonstrated that the maximum concentration of field in the cylinder hole is obtained when the shell has radial and angular permeabilities *μ*_*ρ*_ → ∞ and *μ*_*φ*_ → 0, respectively^30^,^42^. In that case there is no distortion of the field outside the cylinder, so it becomes magnetically undetectable.

We can now explore in the case of a 3D spherical shell the field concentration that corresponds to a non-distortion solution. The magnetic dipole moment *m*^EXT^ in Eq. (2) controls the distortion of the field outside the shell. Depending on its sign, the magnetic field lines will be attracted towards the shell (*m*^EXT^ > 0) or expelled from it (*m*^EXT^ < 0). The solutions of Eq. (2) for *m*^EXT^ = 0 are the cases of non-distortion of the uniform applied field, occurring when


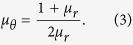


**Figure 1:**
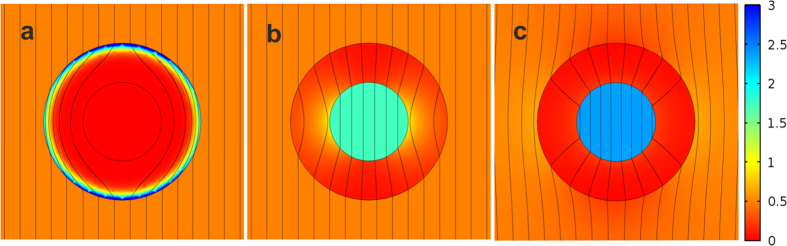
Comparison of spherical shells. Magnetic induction **B** lines and energy density (1/2 **H** · **B**; in colours) when a uniform external magnetic field is applied in the presence of a shell with radii ratio *R*_2_/*R*_1_ = 2 and magnetic permeabilities (**a**) *μ*_*r*_ = 10^4^ and *μ*_*θ*_ = 10^4^, (**b**) *μ*_*r*_ = 10^−4^ and *μ*_*θ*_ = 1/2, and (**c**) *μ*_*r*_ = 10^4^ and *μ*_*θ*_ = 10^−4^.

Considering the permeability relation of Eq. (3) and Eq. (1), one obtains that the magnetic field inside the hole for the non-distortion solutions is


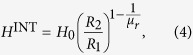


which resembles the analogous relation 
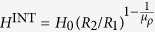
 for a cylindrical shell with permeabilities *μ*_*ρ*_ and *μ*_*φ*_ and inner and outer radii *R*_2_ and *R*_1_, respectively, in a perpendicularly applied magnetic field *H*_0_^30^,^42^. In Eq. (4) the larger the value of *μ*_*r*_, the higher the value of the concentrated field inside the shell. In the limit *μ*_*r*_ → 0 (*μ*_*θ*_ → ∞) the field in the hole is zero (Fig. 1a), i. e. it is a cloaking case, while in the opposite limit *μ*_*r*_ → ∞ (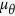 = 1/2), one obtains 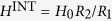 (Fig. 1b).

### Maximum field concentration

We are now ready to tackle the question of whether in the 3D spherical case the non-distortion case (*μ*_*r*_, *μ*_*θ*_) = (∞, 1/2) is the one providing maximum concentrated field in the hole, or if instead there are other solutions providing even more concentration. By analysing Eq. (1) fixing the value of *μ*_*θ*_, the maximum field inside the hole is achieved when *μ*_*r*_ → ∞, and when fixing the value of *μ*_*r*_, the maximum field inside the hole is achieved when *μ*_*θ*_ → 0. Therefore, the absolute maximum is found in the limit *μ*_*r*_ → ∞ and *μ*_*θ*_ → 0 and is


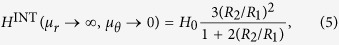


which enhances the applied field by a factor that increases with *R*_2_/*R*_1_. In particular, this enhancement can ideally be made arbitrarily large by making *R*_1_ arbitrarily small. The concentration in this case is larger than the one that can be achieved with a non-distorting spherical shell, 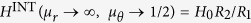 for all possible *R*_2_/*R*_1_ values. This is fundamentally different from the cylindrical case, for which the non-distorting shell yields the maximum concentration in the hole.

The differences between the shells with (*μ*_*r*_, *μ*_*θ*_) = (∞, 1/2) and (*μ*_*r*_, *μ*_*θ*_) = (∞, 0) can be seen in Fig. 1b and c. Interestingly, in the case of maximum concentration, the sphere expels some of the magnetic field lines, i.e, it has a diamagnetic behavior.

We thus conclude that a 3D spherical shell with (*μ*_*r*_, *μ*_*θ*_) = (∞, 0) can yield a large concentration of an applied magnetic field in its hole, and that the maximum field concentration is not a non-distortion situation. This maximum field concentration shell is the one that, once appropriately discretized, is experimentally tested below.

### Energy analysis

To further understand the concentration properties for the different 3D spherical shells, we now analyze these cases in terms of their magnetic energy. From the analytic expressions of the magnetic scalar potential in the shell region (see Supplementary Discussion 1), it can be seen that for the maximum concentration shell (*μ*_*r*_, *μ*_*θ*_) = (∞, 0) the magnetic induction 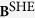 has radial direction and the magnetic field 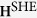 has angular one inside the shell volume. Thus, the energy density in the shell is 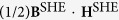 = 0, as happened for the cylindrical shell with maximum field concentration^30^,^42^. Using Eq. (5) one obtains that the total energy inside the hole of the shell in the case of maximum concentration is 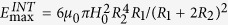. This energy is smaller than the energy that would be in the volume between *r* = 0 and *r* = *R*_2_ without the presence of the shell 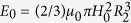, for all radii ratio. Since the total energy inside the shell is zero, having an energy inside the hole smaller than *E*_0_ can only be understood if part of the energy is expelled towards the exterior. Hence, in the case of maximum concentration, part of the energy that would be in the region 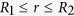 if there was no shell, is moved into the hole of the shell and the rest is placed in the external region, which results in the observed external field distortion (Fig. 1c).

In contrast, for the case (*μ*_*r*_, *μ*_*θ*_) = (∞, 1/2), which is a case of non-distortion of the external field (Fig. 1b), the sum of the energy inside the hole and the energy in the shell is *E*_0_. This energy is larger than the energy concentrated in the spherical hole 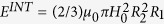. We thus see that not all the energy that was in the shell has been redistributed and placed inside the hole, because part of the energy is kept in the shell (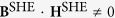), in contrast to the case of a cylindrical shell with the equivalent properties^30^,^42^.

The analytic expressions for the energy in all these cases can be found in Supplementary Discussion 1 and Supplementary Figure S1.

### Field and gradient enhancement for a dipolar source

We have demonstrated that a spherical shell with (*μ*_*r*_, *μ*_*θ*_) = (∞, 0) yields the largest concentration in its hole. One could then use it as a field enhancer to increase the sensitivity of a magnetic sensor by surrounding the sensor with the shell [Eq. (5)]. However, all the results above were derived for a uniform applied field. Interestingly, our results also hold for a non-uniform applied magnetic field. We next analyze the response of a spherical shell to the most typical inhomogeneous field, that of a dipolar source. The details of the derivations in this section can be found in the Supplementary Discussion 2.

Consider a magnetic dipole placed outside the spherical shell at a position 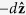 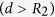 with a magnetic moment 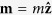. The origin of coordinates is set at the center of the spherical shell.

For a shell with permeabilities (*μ*_*r*_, *μ*_*θ*_ )= (∞, 0) the magnetic field at its center, *r* = 0, is


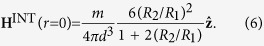


We can compare this value with the magnetic field at *r* = 0 created by the dipole, 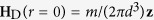, to find that by using the spherical concentrator the magnetic field at *r* = 0 is increased by a factor,


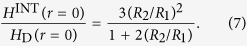


Actually, this enhancement factor at *r* = 0 is exactly the same as the shell concentration ratio for a uniform applied field in all the volume *r* < *R*_1_ [Eq. (5)].

The derivative of the *z* component of the field with respect to *z* at *r* = 0 when using a spherical concentrator is also increased, in this case by a factor,


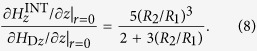


Interestingly, when considering the case of an external dipole with its magnetic moment not pointing to the sphere but perpendicular to it, it can be demonstrated that field and its derivative along *z* are enhanced by the same factors as for the dipole pointing radially [Eqs. (7) and (8)]. Therefore, the same relations will thus be valid for an arbitrary orientation of the dipole. Current applications that use magnetic sensors to find the strength and position of an external source can then benefit from the use of our metamaterial shells since both field and gradient are enhanced by known factors. This is especially important in applications such as geophysical explorations^43^ or magnetoencephalography^20^.

### Shell design and discretization

We next experimentally demonstrate that our spherical shells can enhance the sensitivity of actual magnetic sensors, with a proof-of-concept realization. We have seen above that the spherical shell that concentrates the field the most has (*μ*_*r*_, *μ*_*θ*_) = (∞, 0). This case is chosen for our demonstration, not only because it provides maximum concentration but also because it is more feasible to construct than other options, like the case of maximum concentration for non-distortion, (*μ*_*r*_, *μ*_*θ*_) = (∞, 1/2), which involves intermediate (neither very small nor very large) values of *μ*_*θ*_.

There are different possibilities to emulate the spherical shell concentrator. In previous works in 2D cylindrical geometry^30^,^42^,^44^ shells for concentrating magnetic fields (having *μ*_*ρ*_ → ∞ in the radial direction and *μ*_*ϕ*_ → 0 in the angular one) were devised and constructed using metamaterials composed of alternated sheets of soft ferromagnetic and superconducting material radially aligned. The ferromagnetic layers (FM, with high permeability) provided the required large *μ*_*ρ*_ values whereas the superconducting (SC, with low permeability) - or simply conducting in ref. 44- layers prevented undesired angular components of field.

**Figure 2:**
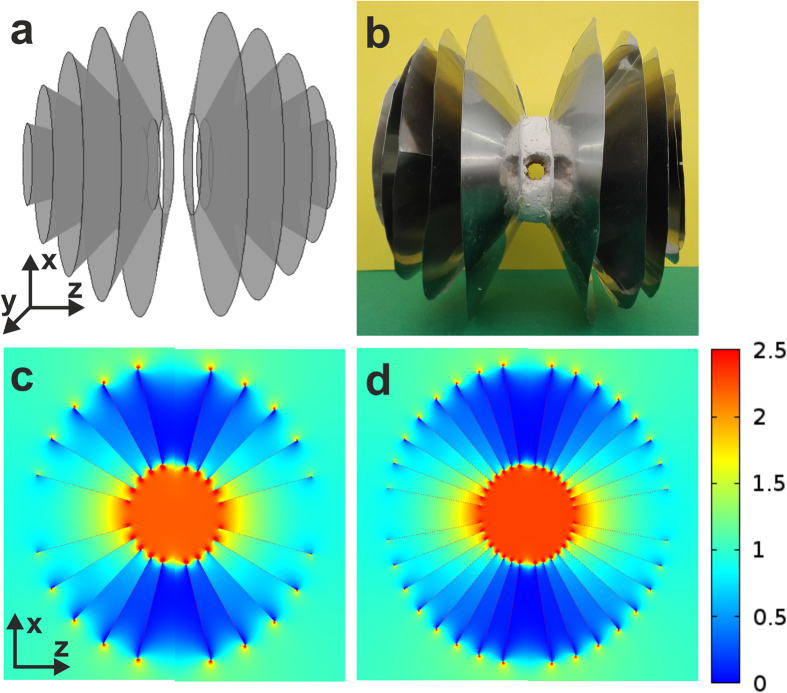
Shell discretizations. (**a**) 3D image of a funnel discretization of the spherical shell with (*μ*_*r*_ → ∞, *μ*_*θ*_ → 0). (**b**) View of the actual device. Finite-element calculations of *B*_*z*_/*B*_0_ considering 5 funnels (**c**) and 8 funnels (**d**) placed on each side of the sphere.

Many actual magnetic sensors measure only one component of the field^1^. For simplicity, we focus our experimental realization on such a case, by designing a discretization of our 3D spherical concentrating shell that is anisotropic, i. e., it mainly concentrates the field coming from one direction. We discretize the shell as a metamaterial composed of concentric and equidistant funnels made of soft ferromagnetic material (Fig. 2 and Methods).

We have numerically studied the field concentration for different numbers of funnels by finite-elements simulations (see Methods). Assuming an ideal anisotropic shell with a radii ratio *R*_2_/*R*_1_ = 3 in a uniform applied magnetic field 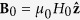, the field is concentrated as 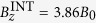 [Eq. (5)]. The simulation for shells consisting of 5 and 8 funnels on each side are shown in Fig. 2c and d, respectively. Placing 5 funnels on each side of the spherical shell yields a concentration factor of 2.22 at the center. This factor is increased by only about 4% when placing 8 + 8 funnels, so we construct our shell with the simpler case of 5 + 5 funnels.

In order to provide a more feasible realization, we have not included any superconducting funnel. The finite-element simulations show that if 5 + 5 ideal superconducting funnels were added alternated with the ferromagnetic ones, then the concentration ratio would be increased to 3.33. However, the use of superconductors would require complex cryogenics, whereas our device operates at room temperature under normal conditions.

### Experimental results

**Figure 3:**
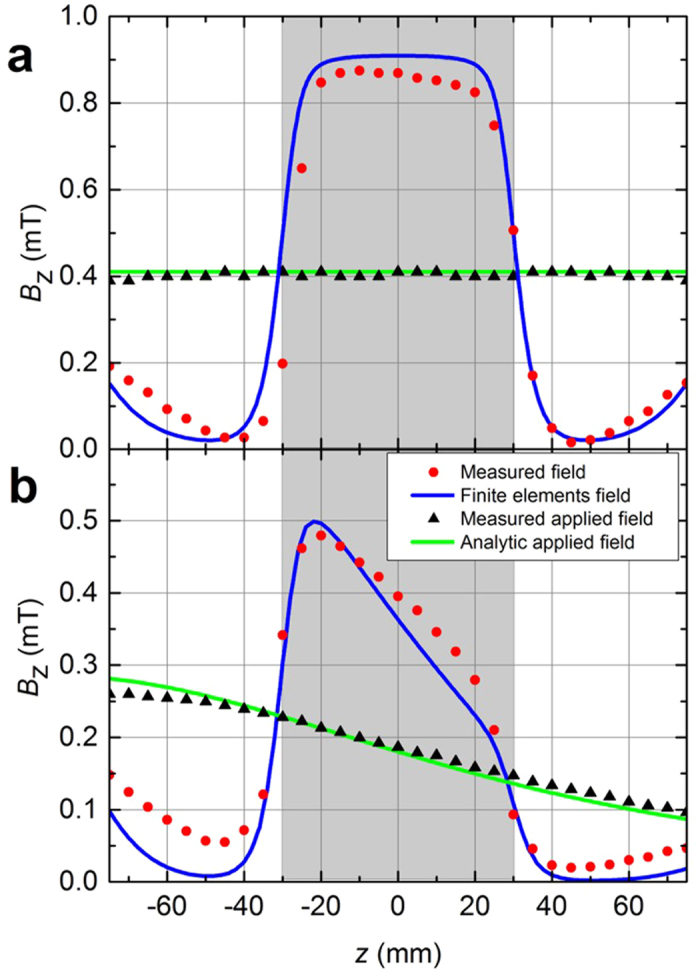
Experimental results for an applied dc field. Measurements (red circles) and finite-elements simulations (blue lines) of the *z* component of the magnetic induction **B** along the *z*-axis when placing the spherical shell in (**a**) a uniform field created by a pair of Helmholtz coils and (**b**) a non-uniform field created by a single coil. Measured (black triangles) and analytic (green lines) values of the applied fields *B*_*z*_ are also depicted. The shadowed regions indicate the shell hole. In the experimental data, errors bars fit within the symbol size.

We first fed a pair of Helmholtz coils with a dc current *I* = 0.5 A to provide a uniform magnetic field in the *z* direction, *B*_0_ = 0.41 ± 0.01 mT. We place the spherical shell between the coils to measure the 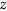-component of **B** along the *z*-axis with a Hall probe [Fig. 3a]. The measured field concentration ratio 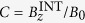 at the center (*z* = 0) of the spherical shell is approximately 2.12; the field is rather uniform in the concentration region except in the zones close to the funnels. Results are in agreement with the finite-element simulations (blue line in Fig. 3a), taking into account that the funnels are not perfectly cut and positioned in the setup.

Since the discretization of the metamaterial shell is optimized to concentrate the field coming from a given direction (its axis), it is interesting to explore the concentrated fields when applying a uniform field in other directions. In the Supplementary Figure S2a we show the experimentally measured angle of the field concentrated at the center of the spherical shell, 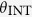, as a function of the angle of the applied field, 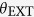. It can be observed that 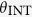 is always lower than 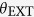, so that **B** at the center tends to bend towards the *z* direction, because the discretization is designed to concentrate fields in this direction (*θ* = 0). This is illustrated in Supplementary Figure S2b, where the measured *B*_*z*_ is shown to be amplified by the shell the same factor for all angles, whereas *B*_*x*_ is not only not amplified by the shell, but reduced.

We next experimentally study the concentration effect for non-uniform fields, for which we connect only one of the Helmholtz coils. Following the same procedure and feeding a steady current of 0.5 A in the coil, we measure *B*_*z*_ along the *z* direction with and without the concentrating shell [Fig. 3b]. Good agreement between experiments and finite-element calculations is found also in this case. *B*_*z*_ decreases linearly in the hole region. The experimental enhancement of *B*_*z*_ right at the center is approximately $C$ = 2.12. We observe that not only the field but its gradient in the hole is enhanced as well.

**Figure 4:**
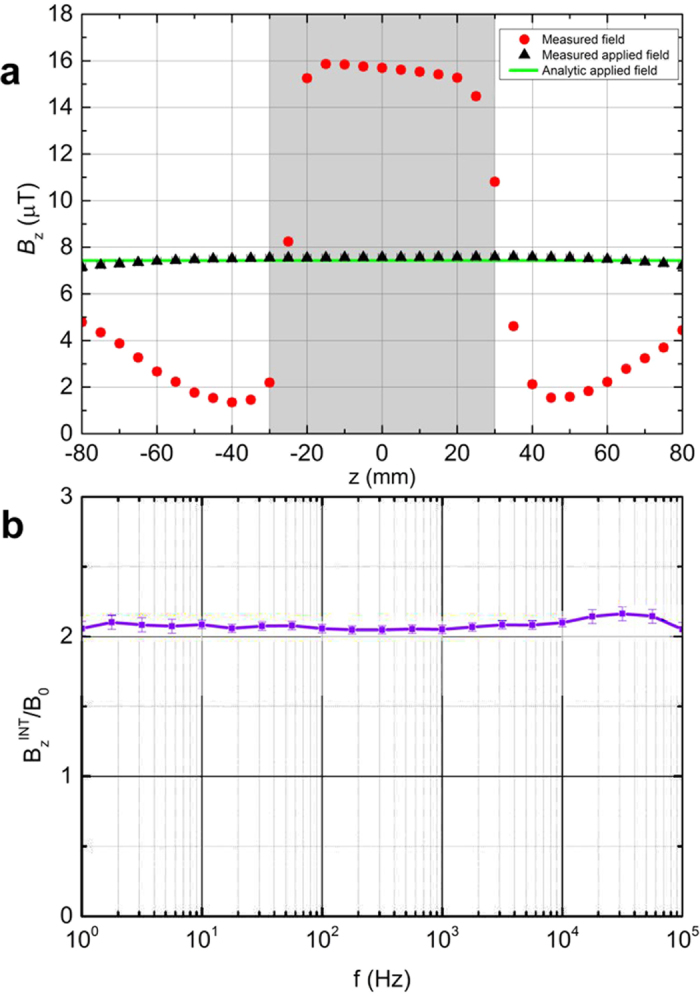
Experimental results for an applied ac field. (**a**) Measurements (red circles) of the *z*-component of the magnetic induction **B** (rms value) along the *z*-axis when placing the spherical shell in a uniform field created by a pair of Helmholtz coils excited by a sinusoidal current at at frequency *f* = 200 Hz. Measured (black triangles) and analytic (green lines) values of the applied field *B*_*z*_ are also depicted. The shadowed region indicates the shell hole. (**b**) Concentration ratio at the center of the shell 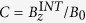 as a function of the frequency *f*.

Our metamaterial shells maintain the field concentration properties also when the applied field is sinusoidally varying with time. In Fig. 4a we show the measured rms amplitude of *B*_*z*_ along the *z* axis when a sinusoidal current 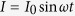 is fed in the Helmholtz coils (*I*_0_ = 10 mA rms and the frequency 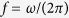 is 200 Hz; the field generated at the center is 7.43 *μ*T rms). The overall pattern is very similar to the dc results (Fig. 3a). Actually, the measured concentration ratio *C* at the center is basically constant with varying frequency for 5 orders of magnitude (from 1 Hz to 100 kHz), as shown in Fig. 4b. Interestingly, besides concentrating the field, the shell is not modifying the phase of the applied field, as shown in Supplementary Figure S3, except for frequencies larger than 10 kHz, at which ohmic losses probably develop because of the conductivity of the funnels. Similarly as previous experimental results^35^,^44^,^45^,^46^,^47^, we confirm the validity of our magnetic metamaterial, originally derived for the dc case, in the quasistatic region of low frequency electromagnetic waves.

## Discussion

We have presented the theory and a proof-of-concept demonstration of a general way to increase the sensitivity of a magnetic sensor by surrounding it with a spherical shell that concentrates the magnetic field in the vicinity of the sensor. The practical realization is only one among many different implementations that can be made. The particular realization studied in this work is chosen for its practical feasibility. After an adequate optimization process, the scale of our metamaterial could be reduced and the concentration factor could be largely increased, for example, by decreasing the inner radius of the shell [Eq. (5)], something feasible taken into account the very small size of many current magnetic sensors.

Many of the most important applications of magnetic sensors involve detecting tiny magnetic fields, in fields like biomedecine, oil prospection, space exploration or geomagnetism^16^,^17^,^18^,^19^. In this limit of very small fields, our assumption that soft ferromagnetic materials have a linear non-hysteretic behavior should be well fulfilled, so our ideas can be readily applied. When concentrating larger fields, non-linear and hysteretic effects in the ferromagnetic parts should be taken into account.

Our proof-of-concept realization involves only ferromagnetic parts arranged in a particular discretization. Numerical simulations show that the addition of alternated superconducting parts would increase the concentration efficiency, at the cost of requiring cryogenics to cool the superconductors below their critical temperature. Actually, in setups involving SQUID sensors, for example, the same cryogenic environment used for the sensors could be used for cooling the concentration shells.

We have experimentally realized a discretization that involves a preferential direction of the applied field. Other concentrators that preserve the spherical symmetry could be realized (e. g. with ferromagnetic spires radially aligned); they would be able to enhance an applied field coming from an arbitrary direction.

In summary, we have theoretically predicted and experimentally demonstrated that the magnetic field in the sensor area is enhanced by our metamaterial shell and that, when the applied field is non-uniform, field gradient is increased as well. The field enhancement is maintained for time-dependent fields upto a frequency of 100 kHz. We thus conclude that magnetic metamaterials can represent a new paradigm for increasing the sensitivity of many kinds of magnetic sensors.

## Methods

For the concentrating spherical shell we build a system consisting of 5 concentric and equidistant funnels (on each side), supported with a non-magnetic material which has openings along the *z* and *x* directions, to emulate a spherical shell with inner and outer radii *R*_1_ = 30 mm and *R*_2_ = 90 mm, respectively (see Fig. 2). Each funnel is made from nickel alloy foil (mu-metal) 0.1 mm thick. Its relative permeability is nominally 8 × 10^4^ at a field of 4 mT, with very low anisotropy, and its resistivity is around 50 *μ*Ω·cm.

The discretized sphere is placed equidistantly between a pair of Helmholtz coils, which provide a uniform field in the sphere region. The coils have a self-inductance of 18.1 mH and a series parasitic resistance of 2.4 Ω. The sphere is supported with a structure made of non-magnetic material.

For the dc measurements, an adjustable dc current is supplied in the coils (only one coil is energized in the non-uniform field measurements). Static dc field measurements are performed by a Hall probe Arepoc.

For the ac measurements, an ac signal at a fixed frequency (from 1 Hz to 100 kHz) is applied to the Helmholtz coils. The field is measured by the voltage induced in a hand-made coil with 110 turns and an average area of 1.61 × 10^−5^ m^2^. The voltage is measured with a Signal Recovery 7265 DSP Lock-In Amplifier and later converted through the following equation,


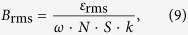


where 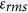 is the coil measured voltage, *ω* is the angular frequency, *N* is the number of turns, *S* is the average area and *k* is a conversion variable that depends on the operating frequency.

Finite-elements calculations are performed using the AC/DC module of the Comsol Multiphysics software. The value of the relative permeability used for funnels simulating the experiments is 8 × 10^4^, and we have assumed the material isotropic.

## Additional Information

**How to cite this article:** Navau, C. *et al*. Enhancing the sensitivity of magnetic sensors by 3D metamaterial shells. *Sci. Rep.* **7**, 44762; doi: 10.1038/srep44762 (2017).

**Publisher's note:** Springer Nature remains neutral with regard to jurisdictional claims in published maps and institutional affiliations.

## Supplementary Material

Supplementary Figures and Discussions
