# Supplementary material for: Enhancing the sensitivity of magnetic sensors by 3D metamaterial shells
Source: Sci Rep. 2017 Mar 17;7:44762. doi: 10.1038/srep44762 (PMC5355979; doi:10.1038/srep44762)
Supplement: Supplementary Figures and Discussions [file srep44762-s1.pdf]

Supplementary Information for 'Enhancing the sensitivity of magnetic sensors by 3D metamaterial shells'

Carles Navau[1], Rosa Mach-Batlle[1], Albert Parra[1],
Jordi Prat-Camps[2], Sergi Laut[1], Nuria Del-Valle[1] and Alvaro Sanchez[1]
[1] *Departament de Física, Universitat Autònoma de Barcelona, 08193 Bellaterra, Barcelona, Catalonia, Spain*
[2] *Institute for Quantum Optics and Quantum Information of the Austrian Academy of Sciences, A-6020 Innsbruck, Austria*
Institute for Theoretical Physics, University of Innsbruck, A-6020 Innsbruck, Austria

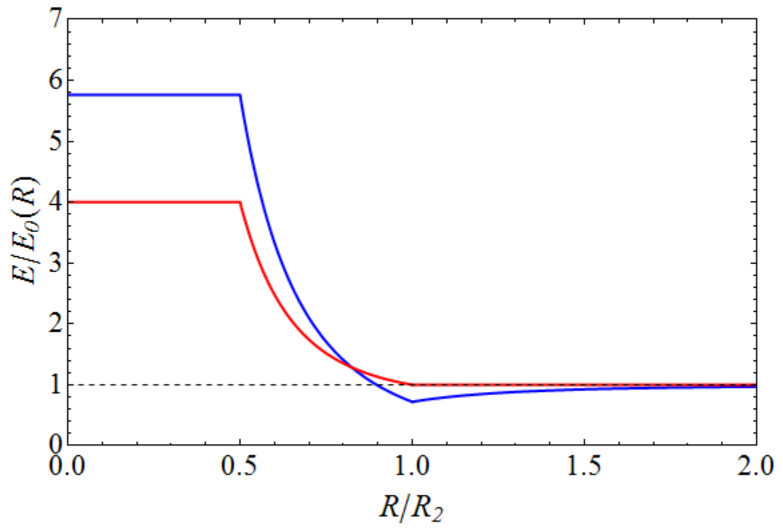

Supplementary Figure S1: Energy distribution. Normalized energy inside a sphere of radius R as a function of the radius R normalized to R_2 for the cases: (black dashed) when no shell is present, (blue) when $(\mu_r, \mu_\theta) = (\infty, 0)$ and (red) when $(\mu_r, \mu_\theta) = (\infty, 1/2)$. The radii ratio of the shell is $\gamma = R_1/R_2 = 0.5$.

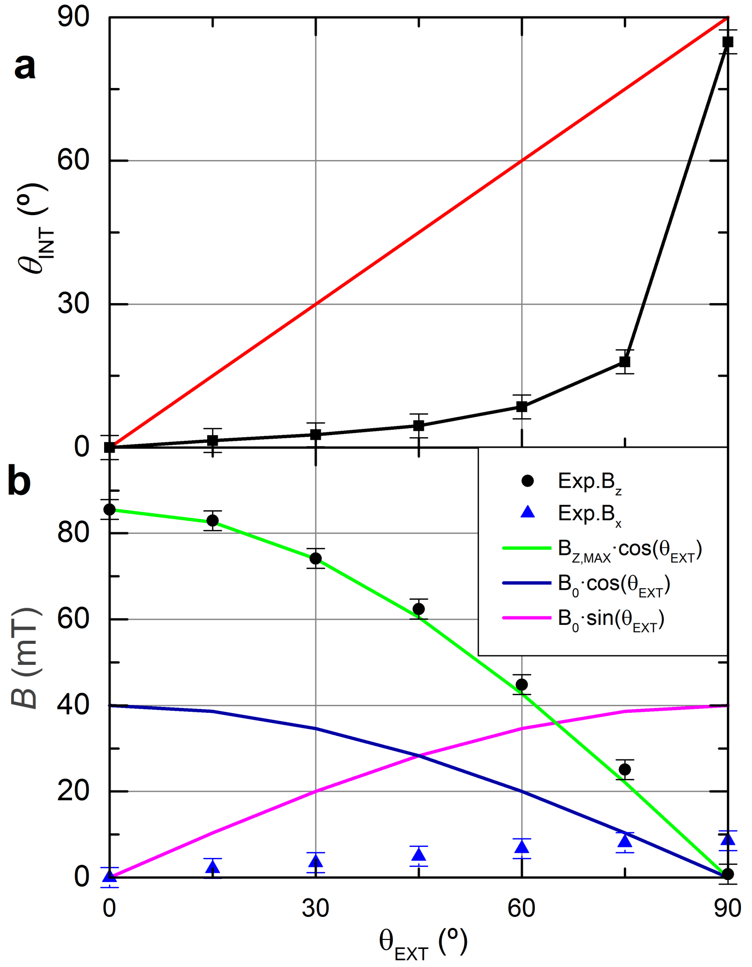

Supplementary Figure S2: Measurements for different applied field directions. (a) Angle of the magnetic induction **B** at the center of the shell (obtained from measurements of B_z and B_x, see below), θ_{INT}, as a function of the angle of the applied magnetic field, θ_{EXT} (black symbols). Black line is a guide to the eye. Red line indicates the relation $\theta_{\text{INT}} = \theta_{\text{EXT}}$ as a reference. (b) Measured B_z (black circles) and B_x (blue triangles) at the center of the shell as a function of the angle of the applied magnetic field θ_{EXT}. B_z follows a cosine dependence (green line) larger than the values of the z-component of the applied field $B_{0,z}$ (blue line) for all angles, whereas B_x is lower than the x-component of the applied field $B_{0,x}$ (magenta line). All angles are defined with respect to the z-axis.

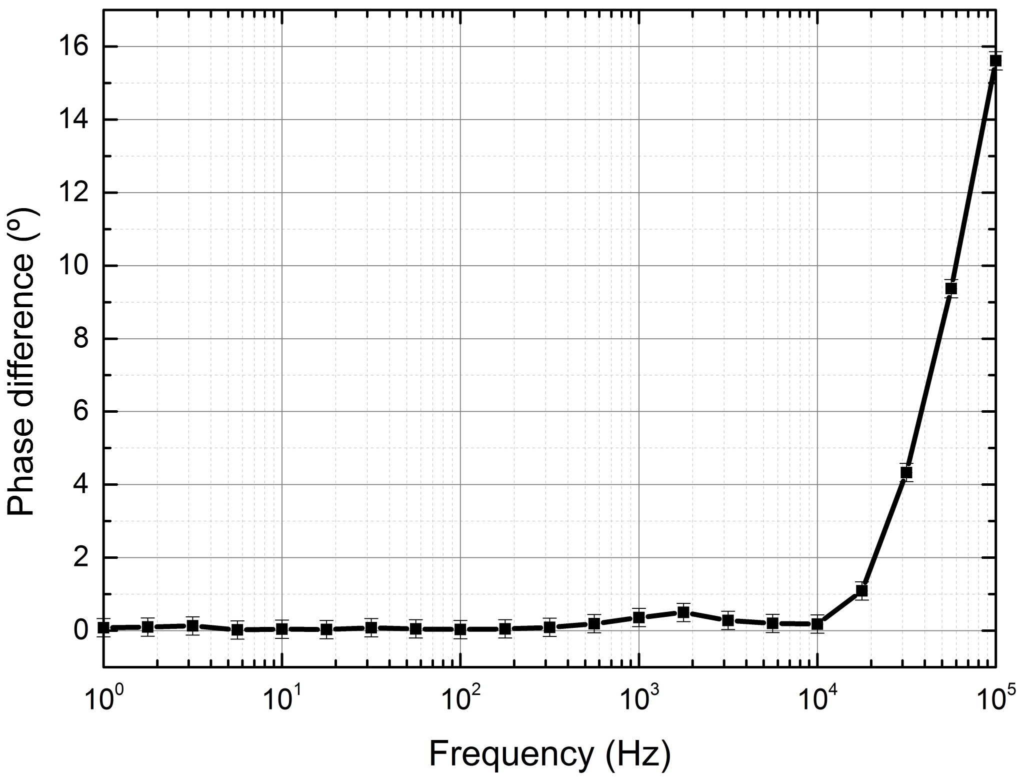

Supplementary Figure S3: Phase difference between the measured and the applied field. Measured phase difference between the measured and applied field as a function of frequency corresponding to the measurements in Fig. 4b of the article. Line is a guide to the eye.

Supplementary Discussion 1: Magnetic field spherical concentrator in a uniform applied magnetic field

In order to study the magnetic concentration properties of a spherical shell in a uniform applied magnetic field, we derive the analytic solutions of the magnetostatic Maxwell equations. Consider a spherical shell of inner radius R_1 and outer radius R_2, centered at $r = 0$, made of a linear, homogeneous and anisotropic magnetic material. The shell is characterized by its polar, azimuthal and radial relative permeabilities, μ_θ, μ_φ and μ_r, such that $B_\theta = \mu_\theta\mu_0 H_\theta$, $B_\varphi = \mu_\varphi\mu_0 H_\varphi$ and $B_r = \mu_r\mu_0 H_r$, being $B_{r,\theta,\varphi}$ and $H_{r,\theta,\varphi}$ the radial, polar and azimuthal components of the magnetic induction $\mathbf{B}$ and the magnetic field $\mathbf{H}$, respectively, and μ_0 the permeability of free space. We choose $\mu_\varphi = \mu_\theta$ for simplicity. A uniform magnetic field $\mathbf{H_0}$ is applied in the z direction. Since there are not free currents in our system, $\nabla \wedge \mathbf{H} = 0$, the magnetic field can be written in terms of a magnetic scalar potential ϕ as $\mathbf{H} = -\nabla\phi$ everywhere in space. In the shell itself (SHE: $R_1 < r < R_2$), the scalar potential must fulfill the equation

$$\nabla^2\phi^{\text{SHE}} = \frac{\partial}{\partial r}\left(r^2\frac{\partial\phi^{\text{SHE}}}{\partial r}\right) + \frac{1}{\sin\theta}\frac{\mu_\theta}{\mu_r}\frac{\partial}{\partial\theta}\left(\sin\theta\frac{\partial\phi^{\text{SHE}}}{\partial\theta}\right) = 0, \tag{S1}$$

while in the interior hole region (INT: $r \leq R_1$) and in the external region (EXT: $r \geq R_2$) the scalar potential should satisfy,

$$\nabla^2\phi^{\text{INT,EXT}} = \frac{\partial}{\partial r}\left(r^2\frac{\partial\phi^{\text{INT,EXT}}}{\partial r}\right) + \frac{1}{\sin\theta}\frac{\partial}{\partial\theta}\left(\sin\theta\frac{\partial\phi^{\text{INT,EXT}}}{\partial\theta}\right) = 0. \tag{S2}$$

The general solutions of these equations, taking into account that ϕ must be finite when $r \to 0$ and tend to $-H_0 r\cos\theta$ when $r \to \infty$, can be written as

$$\phi^{\text{INT}} = \sum_{n=1}^{\infty} a_n^{\text{INT}} r^n \cos(n\theta + \alpha_n^{\text{INT}}), \tag{S3}$$

$$\phi^{\text{SHE}} = \sum_{n=1}^{\infty} a_n^{\text{SHE}} r^{1/2(-1-\sqrt{1+4n(1+n)\mu_\theta/\mu_r})}\cos(n\theta + \alpha_n^{\text{SHE}}) + \sum_{n=1}^{\infty} b_n^{\text{SHE}} r^{1/2(-1+\sqrt{1+4n(1+n)\mu_\theta/\mu_r})}\sin(n\theta + \beta_n^{\text{SHE}}), \tag{S4}$$

$$\phi^{\text{EXT}} = \sum_{n=1}^{\infty} b_n^{\text{EXT}} r^{-n-1}\sin(n\theta + \beta_n^{\text{EXT}}) - H_0 r\cos\theta. \tag{S5}$$

Applying magnetostatic boundary conditions (continuity of the radial component of $\mathbf{B}$ and of the tangential component of $\mathbf{H}$) it follows that $\alpha_1^{\text{INT}} = \alpha_1^{\text{SHE}} = 0$, $\beta_1^{\text{SHE}} = \beta_1^{\text{EXT}} = \pi/2$, $a_n^{\text{INT}} = a_n^{\text{SHE}} = b_n^{\text{SHE}} = b_n^{\text{EXT}} = 0$ when $n > 1$, and

$$a_1^{\text{INT}} = H_0\frac{-6\mu_r\alpha(R_2/R_1)^{(3+\alpha)/2}}{F + G(R_2/R_1)^\alpha}, \tag{S6}$$

$$a_1^{\text{SHE}} = H_0\frac{-3(-2 - \mu_r + \mu_r\alpha)R_2^{(3+\alpha)/2}}{F + G(R_2/R_1)^\alpha}, \tag{S7}$$

$$b_1^{\text{SHE}} = H_0\frac{-3(2 + \mu_r + \mu_r\alpha)R_2^{(3+\alpha)/2}R_1^{-\alpha}}{F + G(R_2/R_1)^\alpha}, \tag{S8}$$

$$b_1^{\text{EXT}} = H_0\frac{-2(-1 - \mu_r + 2\mu_\theta\mu_r)R_2^3(1 - (R_2/R_1)^\alpha)}{F + G(R_2/R_1)^\alpha}, \tag{S9}$$

where $F = -4 - \mu_r - 4\mu_r\mu_\theta + 3\mu_r\alpha$, $G = 4 + \mu_r + 4\mu_r\mu_\theta + 3\mu_r\alpha$, and $\alpha^2 = 1 + 8\mu_\theta/\mu_r$.

From these equations two important results arise: (i) the field inside the spherical hole is a uniform field in the direction of the applied magnetic field with magnitude $H^{\text{INT}} = -a_1^{\text{INT}}$ and (ii) the field created by the sphere at the exterior region corresponds to the field of a dipole centered at the origin of coordinates with magnetic moment $\mathbf{m} = 4\pi b_1^{\text{EXT}}\hat{\mathbf{z}}$.

A. Energy analysis

It is demonstrated in the main text that the maximum concentration of magnetic field inside the hole of a shell is achieved when using a spherical shell with permeabilities $\mu_r \to \infty$ and $\mu_\theta \to 0$. Since the coefficient $b_1^{\rm EXT}$ is not zero for these permeabilities, Eq. (S9), this shell distorts the external magnetic field. Another interesting spherical shell, which concentrates the magnetic field inside its hole while being magnetically undetectable is obtained when considering the permeabilities $\mu_r \to \infty$ and $\mu_\theta \to 1/2$.

In this section we compare the energy in a sphere of radius R for a shell with $(\mu_r, \mu_\theta) = (\infty, 0)$, $E_{\rm max}$, to the energy in the same region for a shell with $(\mu_r, \mu_\theta) = (\infty, 1/2)$, $E_{\rm nd}$. These energies are normalized by E_0, which is the energy that there would be in the region $r < R$ if no shell was present. The analytic expressions for these energies are

$$\frac{E_{\rm max}(R)}{E_0(R)} = \begin{cases} \frac{9}{\gamma^2(2+\gamma)^2} & \text{if } R < R_1 \\ \frac{9\gamma}{(2+\gamma)^2}\left(\frac{R_2}{R}\right)^3 & \text{if } R_1 \le R \le R_2 \\ \frac{9\gamma}{(2+\gamma)^2}\left(\frac{R_2}{R}\right)^3 + \left(1-\left(\frac{R_2}{R}\right)^3\right)\left[\left(\frac{R_2}{R}\right)^3\left(\frac{\gamma-1}{2+\gamma}\right)^2+1\right] & \text{if } R_2 < R \end{cases}$$

$$\frac{E_{\rm nd}(R)}{E_0(R)} = \begin{cases} \frac{1}{\gamma^2} & \text{if } R < R_1 \\ \gamma\left(\frac{R_2}{R}\right)^3 + 1 - \gamma\frac{R_2}{R} & \text{if } R_1 \le R \le R_2 \\ 1 & \text{if } R_2 < R \end{cases}$$

where $\gamma = R_1/R_2$.

In Supplementary Figure S1, $E_{\rm max}(R)/E_0(R)$ and $E_{\rm nd}(R)/E_0(R)$ are plotted as a function of the radius of the sphere normalized by R_2. It can be observed that $E_{\rm max}(R)$ is larger than $E_{\rm nd}(R)$ in the hole, and also that $E_{\rm max}(R)$ is different from $E_0(R)$ for $R > R_2$.

Supplementary Discussion 2: Field and gradient enhancement for a dipolar source

In this section the analytic solutions of Maxwell equations for a dipolar source are derived to analyse how a spherical shell can concentrate a non-uniform magnetic field. Consider a dipole placed at $-d\hat{\mathbf{z}}$. The scalar magnetic potential in terms of the spherical coordinates centered at the origin r, θ, and φ, is

$$\phi_D = \frac{m\left[r\mathrm{sin}\theta\mathrm{sin}\theta''\mathrm{cos}(\varphi-\varphi'')+(r\mathrm{cos}\theta+d)\mathrm{cos}\theta''\right]}{4\pi(r^2+d^2+2dr\mathrm{cos}\theta)^{3/2}}, \tag{S10}$$

where θ'' and φ'' are the spherical angles of the magnetic moment of the dipole $\mathbf{m}$.

A. Dipole pointing towards the center of a spherical shell

We consider a spherical shell of external radius R_2 and internal radius R_1. Its magnetic permeability is homogeneous, linear and anisotropic, being μ_r in the radial direction, μ_φ in the azimuthal direction and μ_θ in the polar direction. For simplicity, we choose $\mu_\varphi = \mu_\theta$.

Consider a magnetic dipole placed outside the spherical shell, at $-d\hat{\mathbf{z}}$ ($d > R_2$), with magnetic moment $\mathbf{m} = m\hat{\mathbf{z}}$. In this case $\theta'' = 0$ and Eq. (S10) becomes

$$\phi_D = \frac{m}{4\pi}\frac{r\mathrm{cos}\theta+d}{(r^2+d^2+2dr\mathrm{cos}\theta)^{3/2}}. \tag{S11}$$

The magnetic potential of Eq. (S11) can be written in terms of a sum of Legendre Polynomials, $P_n(\mathrm{cos}\theta)$, as

$$\phi_D = \begin{cases} m/(4\pi d^2)\sum_{n=0}^{\infty}(n+1)(-1)^n\,(r/d)^n\,P_n(\mathrm{cos}\theta) & \text{if } r\le d,\\ m/(4\pi d^2)\sum_{n=0}^{\infty}n(-1)^{n+1}\,(d/r)^{n+1}\,P_n(\mathrm{cos}\theta) & \text{if } r> d.\end{cases} \tag{S12}$$

Since there are no free currents in the system, $\nabla \wedge \mathbf{H} = \mathbf{0}$, the magnetic field can be written in terms of a magnetic scalar potential ϕ in the whole space, $\mathbf{H} = -\nabla\phi$. By taking into account $\nabla\cdot\mathbf{B} = 0$, $B_r = \mu_r H_r$ and $B_\theta = \mu_\theta H_\theta$ we obtain the equations that the scalar potential should satisfy in the whole space [Eqs. (S1) and (S2), since the problem is azimuthally symmetric]. The general solutions of these equations inside the hole (INT: $r \le R_1$), inside the shell (SHE: $R_1 < r < R_2$) and in the external region (EXT: $r \ge R_2$) can be written as

$$\phi^{\mathrm{INT}} = \sum_{n=0}^{\infty} a_n r^n P_n(\mathrm{cos}\theta), \tag{S13}$$

$$\phi^{\mathrm{SHE}} = \sum_{n=0}^{\infty} b_n r^{\frac{1}{2}\left(-1-\sqrt{1+4n(1+n)\mu_\theta/\mu_r}\right)} P_n(\mathrm{cos}\theta) + \sum_{n=0}^{\infty} c_n r^{\frac{1}{2}\left(-1+\sqrt{1+4n(1+n)\mu_\theta/\mu_r}\right)} P_n(\mathrm{cos}\theta), \tag{S14}$$

$$\phi^{\mathrm{EXT}} = \sum_{n=0}^{\infty} d_n r^{-n-1} P_n(\mathrm{cos}\theta) + \phi_D, \tag{S15}$$

where we have considered that the scalar potential should be finite when $r \to 0$ and tend to the applied potential, ϕ_D, Eq. (S12), when $r \to \infty$.

At this point, we apply magnetostatic boundary conditions. The radial component of the magnetic induction $\mathbf{B}$ and the tangential component of $\mathbf{H}$ must be continuous at $r = R_1$ and $r = R_2$ in order to satisfy $\nabla\cdot\mathbf{B} = 0$ and $\nabla \wedge \mathbf{H} = 0$, respectively. The obtained coefficients are,

$$a_n = \frac{m(-1)^n(n+1)(2n+1)qR_2^{(q+1)/2}\mu_r(R_2/d)^n R_1^{(q-1)/2-n}}{2\pi d^2\left(\mu_r\left(R_1^q\left(2n\left(q-(n+1)\mu_\theta\right)+q-1\right)+R_2^q\left(2n\left(\mu_\theta+n\mu_\theta+q\right)+q+1\right)\right)-2n(n+1)\left(R_1^q-R_2^q\right)\right)}, \tag{S16}$$

$$b_n = \frac{m(-1)^n(n+1)(2n+1)R_1^q R_2^{(q+1)/2}(R_2/d)^n\left(2n - q\mu_r + \mu_r\right)}{4\pi d^2\left(\mu_r\left(R_1^q\left(2n\left(\mu_\theta + n\mu_\theta - q\right) - q + 1\right) - R_2^q\left(2n\left(\mu_\theta + n\mu_\theta + q\right) + q + 1\right)\right) + 2n(n+1)\left(R_1^q - R_2^q\right)\right)}, \tag{S17}$$

$$c_n = \frac{m(-1)^n(n+1)(2n+1)R_2^{(q+1)/2}(R_2/d)^n\left(2n + q\mu_r + \mu_r\right)}{4\pi d^2\left(\mu_r\left(R_1^q\left(2n\left(q - (n+1)\mu_\theta\right) + q - 1\right) + R_2^q\left(2n\left(\mu_\theta + n\mu_\theta + q\right) + q + 1\right)\right) - 2n(n+1)\left(R_1^q - R_2^q\right)\right)}, \tag{S18}$$

$$d_n = \frac{m(-1)^n n(n+1)R_2^{n+1}\left(R_1^q - R_2^q\right)(R_2/d)^n\left(\mu_r\left(\mu_\theta + n\mu_\theta - 1\right) - n\right)}{2\pi d^2\left(\mu_r\left(R_1^q\left(2n\left(q - (n+1)\mu_\theta\right) + q - 1\right) + R_2^q\left(2n\left(\mu_\theta + n\mu_\theta + q\right) + q + 1\right)\right) - 2n(n+1)\left(R_1^q - R_2^q\right)\right)}, \tag{S19}$$

where $q = \sqrt{1 + 4n(1+n)\mu_\theta/\mu_r}$.

When a spherical shell is placed in a uniform applied magnetic field, it creates a dipolar field outside its external surface and a uniform field inside its hole. Interestingly, from Eqs. (S13) - (S15) and Eqs. (S16) - (S19), we see that the response of the spherical shell to a dipolar field is more complex, since the magnetic potential it creates is constituted by infinite terms. The response of a cylindrical shell to a dipolar field could also be understood as a sum of infinite terms. However, that sum was equivalent to that of a non-centered dipole. When considering spherical shells this correspondence is not found.

We would like to see if there is a spherical shell that does not distort the magnetic field created by an external dipole. This would require $d_n = 0$ for all n because, as seen in Eq. (S15), this coefficient indicates how the spherical shell distorts an external magnetic field. For a given value of n, $d_n = 0$ is obtained only when

$$\mu_\theta = \frac{\mu_r + n}{\mu_r(1+n)}, \tag{S20}$$

which depends on n. Therefore, a shell with angular and radial permeabilities fulfilling Eq. (S20) for a given n can cancel at most a single n term of the magnetic scalar potential. Consequently, all spherical shells distort the magnetic field created by a dipole in its outer region.

Extreme spherical concentrator

If we consider the extreme spherical concentrator, $(\mu_r, \mu_\theta) = (\infty, 0)$, the coefficients a_n from Eq. (S16), which give the magnetic field inside the shell's hole, are simplified and can be written as

$$a_n = \frac{m}{4\pi d^2}\frac{(-1)^n(1+n)(1+2n)R_1^{-n}R_2\left(R_2/d\right)^n}{R_2 + n(R_1 + R_2)}. \tag{S21}$$

From Eqs. (S13) and (S21), one obtains that the magnetic field at $r = 0$ and the derivative of its z component with respect to z when a spherical concentrator is used are

$$\mathbf{H}^{\mathrm{INT}}(r=0) = \frac{m}{4\pi d^3}\frac{6\left(R_2/R_1\right)^2}{1 + 2\left(R_2/R_1\right)}\hat{\mathbf{z}}, \tag{S22}$$

$$\left.\frac{\partial H_z^{INT}}{\partial z}\right|_{r=0} = -\frac{m}{2\pi d^4}\frac{15(R_2/R_1)^2}{3 + 2R_1/R_2}. \tag{S23}$$

The magnetic field and gradient at $r = 0$ created by a dipole pointing radially (without the presence of a spherical shell) can be obtained from Eq. (S11) and are $\mathbf{H_D}(r=0) = m/(2\pi d^3)\hat{\mathbf{z}}$ and $(\partial H_{Dz}/\partial z)|_{r=0} = -3m/(2\pi d^4)$, respectively. Hence, using an extreme spherical concentrator the magnetic field and gradient at $r = 0$ are increased by the factors,

$$\frac{H^{\mathrm{INT}}(r=0)}{H_D(r=0)} = \frac{3\left(R_2/R_1\right)^2}{1 + 2\left(R_2/R_1\right)}, \tag{S24}$$

$$\frac{(\partial H_z^{\rm INT}/\partial z)\Big|_{r=0}}{(\partial H_{Dz}/\partial z)\Big|_{r=0}} = \frac{5\,(R_2/R_1)^2}{3+2\,(R_1/R_2)}. \tag{S25}$$

which can be increased by designing a spherical concentrator with larger radii ratio R_2/R_1.

B. Dipole pointing towards the non-radial plane

As in section A, we consider a spherical shell of external radius R_2 and internal radius R_1. Its magnetic permeability is homogeneous, linear and anisotropic, being μ_r in the radial direction, μ_φ in the azimuthal direction and μ_θ in the polar direction. We choose $\mu_\varphi = \mu_\theta$ to simplify.

Considering a magnetic dipole placed outside the spherical shell, at $-d\hat{\mathbf{z}}$ ($d > R_2$), with magnetic moment $\mathbf{m}$ perpendicular to the z direction. In this situation, $\theta'' = \pi/2$ and Eq. (S10) becomes

$$\phi_D = \frac{m}{4\pi}\frac{r\sin\theta\cos(\varphi-\varphi'')}{(r^2+d^2+2dr\cos\theta)^{3/2}}, \tag{S26}$$

One can choose the origin of the x and y axis so that $\varphi'' = \pi/2$ ($\mathbf{m} = m\hat{\mathbf{y}}$). Then, $\cos(\varphi-\varphi'') = \sin\varphi$ and the magnetic potential of Eq. (S11) can be written as

$$\phi_D = \begin{cases} m/(4\pi d^2)\sum_{l=0}^{\infty}(-1)^l\,(r/d)^l\,P_l^1(\cos\theta)\sin\varphi & \text{if } r \le d, \\ m/(4\pi d^2)\sum_{l=0}^{\infty}(-1)^l\,(d/r)^{l+1}\,P_l^1(\cos\theta)\sin\varphi & \text{if } r > d, \end{cases} \tag{S27}$$

where $P_l^1(\cos\theta)$ is the first derivative of the Legendre Polynomials $P_l(\cos\theta)$ with respect to θ.

Since there are not free currents in the system, $\nabla\wedge\mathbf{H} = 0$, the magnetic field can be written in terms of a magnetic scalar potential ϕ in the whole space, $\mathbf{H} = -\nabla\phi$. Using $\nabla\cdot\mathbf{B} = 0$, $B_r = \mu_r H_r$, $B_\varphi = \mu_\varphi H_\varphi$ and $B_\theta = \mu_\theta H_\theta$ one can obtain the equations that the scalar potential should satisfy in the whole space. In the shell itself (SHE: $R_1 < r < R_2$), the scalar potential must fulfill the equation

$$\nabla^2\phi^{\rm SHE} = \frac{\partial}{\partial r}\left(r^2\frac{\partial\phi^{\rm SHE}}{\partial r}\right) + \frac{1}{\sin\theta}\frac{\mu_\theta}{\mu_r}\frac{\partial}{\partial\theta}\left(\sin\theta\frac{\partial\phi^{\rm SHE}}{\partial\theta}\right) + \frac{1}{\sin^2\theta}\frac{\mu_\theta}{\mu_r}\frac{\partial^2\phi^{\rm SHE}}{\partial\varphi^2} = 0, \tag{S28}$$

while in the interior hole region (INT: $r \le R_1$) and in the external region (EXT: $r \ge R_2$) the scalar potential should satisfy

$$\nabla^2\phi^{\rm INT,EXT} = \frac{\partial}{\partial r}\left(r^2\frac{\partial\phi^{\rm INT,EXT}}{\partial r}\right) + \frac{1}{\sin\theta}\frac{\partial}{\partial\theta}\left(\sin\theta\frac{\partial\phi^{\rm INT,EXT}}{\partial\theta}\right) + \frac{1}{\sin^2\theta}\frac{\partial^2\phi^{\rm INT,EXT}}{\partial\varphi^2} = 0. \tag{S29}$$

The general solutions of Eqs. (S28) and (S29) can be written in terms of the m derivative of the Legendre Polynomials as

$$\phi^{\rm INT} = \sum_{l=0}^{\infty}\sum_{m=-l}^{l} a_l^m r^l P_l^m(\cos\theta)\sin\varphi, \tag{S30}$$

$$\phi^{\rm SHE} = \sum_{l=0}^{\infty}\sum_{m=-l}^{l} b_l^m r^{l/2\left(-1-\sqrt{1+4l(l+1)\mu_\theta/\mu_r}\right)} Y_l^m(\theta,\varphi) + \sum_{l=0}^{\infty}\sum_{m=-l}^{l} c_l^m r^{l/2\left(-1+\sqrt{1+4l(l+1)\mu_\theta/\mu_r}\right)} P_l^m(\cos\theta)\sin\varphi, \tag{S31}$$

$$\phi^{\rm EXT} = \sum_{l=0}^{\infty}\sum_{m=-l}^{l} d_l^m r^{-l-1} P_l^m(\cos\theta)\sin\varphi + \phi_D, \tag{S32}$$

where we have assumed that the scalar potential should be finite when $r \to 0$ and tend to the applied potential, ϕ_D, Eq. (S27), when $r \to \infty$.

By imposing magnetic boundary conditions we obtain, $a_l^m = b_l^m = c_l^m = d_l^m = 0 \quad \forall m \neq 1$, and

$$a_l^1 = \frac{m\mu_r(-1)^l(2l+1)qR_2^{(q+1)/2}\left(R_2/d\right)^l R_1^{(q-1)/2-l}}{2\pi d^2\left(\mu_r\left(R_1^q(2l(q-\mu_\theta(l+1))+q-1)+R_2^q(2l(\mu_\theta l+\mu_\theta+q)+q+1)\right)-2l(l+1)\left(R_1^q-R_2^q\right)\right)}, \tag{S33}$$

$$b_l^1 = -\frac{m(-1)^l(2l+1)R_1^qR_2^{(q+1)/2}\left(R_2/d\right)^l\left(\mu_r(-q)+\mu_r+2l\right)}{4\pi d^2\left(\mu_r\left(R_1^q(2l(q-\mu_\theta(l+1))+q-1)+R_2^q(2l(\mu_\theta l+\mu_\theta+q)+q+1)\right)-2l(l+1)\left(R_1^q-R_2^q\right)\right)}, \tag{S34}$$

$$c_l^1 = \frac{m(-1)^l(2l+1)R_2^{(q+1)/2}\left(R_2/d\right)^l\left(\mu_r q+\mu_r+2l\right)}{4\pi d^2\left(\mu_r\left(R_1^q(2l(q-\mu_\theta(l+1))+q-1)+R_2^q(2l(\mu_\theta l+\mu_\theta+q)+q+1)\right)-2l(l+1)\left(R_1^q-R_2^q\right)\right)}, \tag{S35}$$

$$d_l^1 = \frac{m(-1)^l lR_2^{l+1}\left(R_2/d\right)^l\left(\mu_r(\mu_\theta l+\mu_\theta-1)-l\right)\left(R_1^q-R_2^q\right)}{2\pi d^2\left(\mu_r\left(R_1^q(2l(q-\mu_\theta(l+1))+q-1)+R_2^q(2l(\mu_\theta l+\mu_\theta+q)+q+1)\right)-2l(l+1)\left(R_1^q-R_2^q\right)\right)} \tag{S36}$$

where $q = \sqrt{1+4l(1+l)\mu_\theta/\mu_r}$.

As in the case of a dipole pointing in the radial direction, the magnetic field in the external region is distorted by the presence of the spherical shell. One can obtain from Eq. (S36) the required magnetic permeabilities to not distort one of the terms, l, of the magnetic potential, but it does not exist any shell providing non-distortion for all l. The obtained relation between μ_r and μ_θ is equivalent to that of Eq. (S20).

Extreme spherical concentrator

When considering the extreme spherical concentrator, $\mu_r \to \infty$ and $\mu_\theta \to 0$, the coefficient a_l^1, which indicates the magnetic field concentration, becomes

$$a_l^1 = \frac{m}{4\pi d^2}\frac{(-1)^n(1+2n)R_1^{-n}R_2(R_2/d)^n}{R_2+n(R_1+R_2)}. \tag{S37}$$

By looking at the form of the magnetic potential inside the hole, Eq. (S30) one sees that, same as for the case of a dipole pointing radially towards the center of the shell, at $r=0$ only the term $l=1$ of the sum of the magnetic field will be different from 0. The magnetic field at this point is

$$\mathbf{H}^{\mathrm{INT}}(r=0) = -\frac{m}{4\pi d^3}\frac{3(R_2/R_1)^2}{1+2R_2/R_1}\hat{\mathbf{y}}, \tag{S38}$$

where $\hat{\mathbf{y}}$ is the direction of the magnetic moment $\mathbf{m}$ of the dipole we have chosen. Analogously, the derivative of the y component of the magnetic field with respect to z is

$$\left.\frac{\partial H_y^{\mathrm{INT}}}{\partial z}\right|_{r=0} = \frac{m}{4\pi d^4}\frac{15(R_2/R_1)^2}{3+2(R_1/R_2)}. \tag{S39}$$

The magnetic field that the bare dipole would create at $r=0$, according to Eq. (S26) is $-m/(4\pi d^3)\hat{\mathbf{y}}$. Hence, by using the extreme spherical concentrating shell the magnetic field at $r=0$ has been increased by the same factor we obtained considering a dipole with magnetic moment in the radial direction [Eq. (S24)]. The derivative of the H_y component of the magnetic field created by this dipole with respect to z at $r=0$ is $(\partial H_{Dy}/\partial z)|_{r=0} = 3m/(4\pi d^4)$. Therefore, the gradient has also been increased by the shell the same ratio we obtained for a radial dipole [Eq. (S25)].
